# The assumption of safety is being used to justify the rollout of 5G technologies

**DOI:** 10.3389/fpubh.2023.1058454

**Published:** 2023-01-26

**Authors:** Julie E. McCredden, Steven Weller, Victor Leach

**Affiliations:** ^1^Oceania Radiofrequency Scientific Advisory Association Inc. (ORSAA), Scarborough, QLD, Australia; ^2^Centre for Environmental and Population Health, School of Medicine and Dentistry, Griffith University, Brisbane, QLD, Australia

**Keywords:** 5G, EMR, wireless radiation, EMF database, environmental health, conflicts of interest, science communication

## Introduction

The advent of fifth-generation (5G) wireless communication introduces new technology utilizing near-millimeter radiofrequency waves [i.e., with a frequency of 30–300 GHz (mmWaves)]. The long-term effects of these signals on humans and the environment are unknown. Scientific literature reviews investigating biological harm from mmWave usage have concluded … *no in-depth conclusions can be drawn…*[(1), p. 16] and *no confirmed evidence* [(2), p. 601]. Unfortunately, these statements of scientific uncertainty have been used by industry and government advisory bodies to reassure the public of the safety of the 5G rollout. However, the assumption that 5G technologies are safe is not an evidence-based conclusion (3). Why this is so cannot be easily understood from existing summaries or reviews (4). Therefore, this article takes one step back from reviews to the original papers, so as to provide a visible overview of the existing mmWave evidence base. It then examines how the science is being conducted and communicated, finding errors in reasoning that cloud judgements and the subsequent conclusions drawn from the existing research.

## Mapping out the mmWave research landscape

Public policy regarding the safety of electromagnetic fields (EMF) is often formulated from reviews rather than from individual papers, e.g., the recent SCHEER opinion (5). Literature reviews give readers a narrow view of past research, with many papers ignored or removed at the beginning of the review process. It is also possible that quality papers are being omitted in this process (4). Thus, all relevant mmWave research literature is not yet fully transparent to the readership in this field. To help the research community to formulate an initial overview opinion, we have mapped out the broader landscape by making visible the range of biological and health effect topics contained within the mmWave literature (see below). Then, within the main topics investigated, we have made evident the number of studies showing effects vs. the number of studies showing no effects “regardless of the study design, merit, flaws, experimental quality, shortcomings, limitations, or methodological weaknesses” [(6), p. 2]. As such, this opinion piece is not to be considered as a systematic review. However, the papers presented here [listed in Supplementary Table 1 (all >6GHz experimental papers) and (epidemiological papers)] could be used as the basis for future exploration utilizing a more formal systematic review approach.

### Database search for studies on mmWaves and health

Literature reviews investigating EMF typically use several existing information sources, such as PubMed, EMF-portal, and the Institute of Electrical and Electronics Engineers (IEEE). However, these databases cover a much broader range of topics than the bioeffects of electromagnetic radiation, such as medical procedures and accidents, computational models and non-experimental theoretical discussions. To address the need for a focused knowledge collection, the Oceania Radiofrequency Scientific Advisory Association (ORSAA) (7) has developed the ORSAA Database of EMF Bioeffects (ODEB) (8) containing peer-reviewed studies investigating the biological and health effects of electromagnetic fields on humans, animals and the environment.

ODEB^1^ was first established using the entire research database of the Australian Radiation Protection & Nuclear Safety Agency (ARPANSA) and then expanded to incorporate all relevant papers from PubMed and the EMF-portal. ODEB also includes military studies from the 70's, biophysics research from the 80's onwards, and all experimental and epidemiological research from both industry and independent scientists since 2012. ODEB currently comprises over 4,000 peer-reviewed publications and is being continually updated. It is searchable in many different categories including biological effect end-points, exposure parameters, study type etc. When papers are added to the ODEB database, they are screened for relevance. This description of the ODEB collection and its sources has been provided to demonstrate that the database is an adequate resource for the mmWave literature overview described below.

### Investigation limited to below-threshold, mmWave papers

The experimental papers delivering mmWave exposures at or below the ICNIRP limits test whether the current ICNIRP exposure thresholds are adequate to guarantee safety for the public. A literature search was thus performed by requesting from ODEB all papers that used radiofrequencies > 6 GHz and exposure intensities below the International Commission on Non-Ionizing Radiation Protection (ICNIRP); i.e., the 4 W/kg whole-body Specific Absorption Rate (SAR) limit and the 200 W m^−2^ local tissue incident power density limit; [(9), p. 6–8]. The result was a set of 295 papers containing all of the papers in the recent Karipidis et al. mmWave review (10), plus an additional 79 more experimental papers (nine non-English) and 19 more epidemiology papers (five non-English). Given that this paper aims to map out the entire landscape, inclusion of the 14 non-English papers is appropriate.

Including all of these sources, the ODEB search produced a current literature base for mmWave research comprising 238 experimental papers and 57 epidemiology papers [see Supplementary Table 1 (all >6GHz experimental papers) and (epidem papers)]. This is a relatively small knowledge base, given the many combinations of experimental parameters requiring examination, such as frequency, modulation pattern, intensity, exposure duration, and the numerous types of tissues, cells, and biological functions. In comparison with the broader radiofrequency literature, mmWave research constitutes <10% of the knowledge base.

### Main themes

As there are so few experimental studies on the bioeffects of mmWaves, rigorous literature reviews at this point in time are most likely destined to find no strong evidence. Instead, it is instructive to map out the main biological and health categories that have been investigated within the entire collection of studies, for the reasons given above and to help identify focus areas for future research.

**Figure 1:**
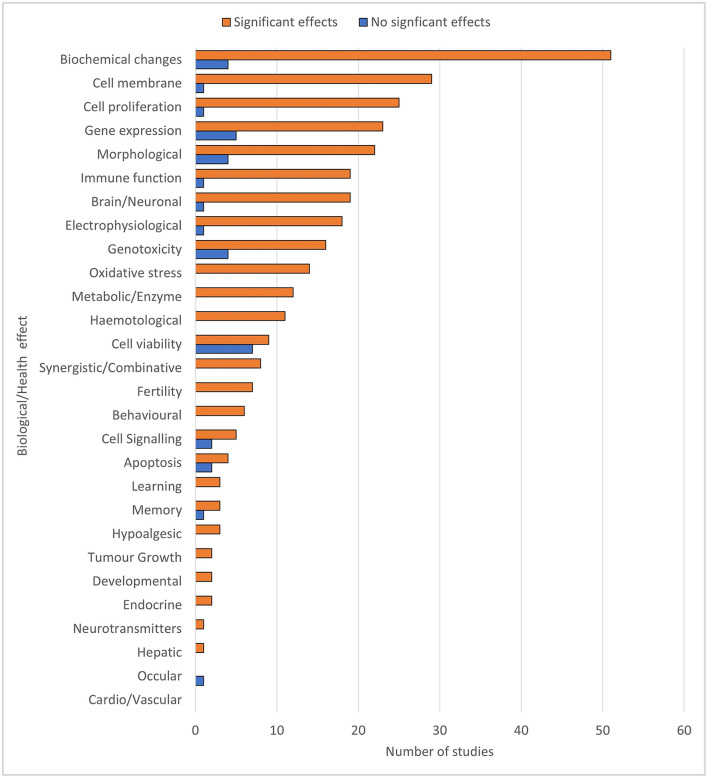
The main biological and health categories present in the mmWave experimental (i.e., *in vitro* and *in vivo*) literature base, and within each category, the number of papers producing effects vs. the number of papers resulting in no significant effects. The total number of studies is greater than the total number of papers because any given paper may have conducted more than one study and investigated more than one biological effect.

Experimental papers emerging from the ODEB literature search (previously described) were automatically classified into their main biological and health categories. Within these, the number of studies showing significant effects and the number of studies showing no significant effects were tabled. Four papers with uncertain effects [i.e., where outcomes were not reported, or conclusions were qualified (8)] were excluded. The results for the experimental studies are summarized in Figure 1 below.

Figure 1 shows that the mmWave experimental studies cover a wide range of bioeffects. Furthermore, for most of the categories in Figure 1, from *biochemical* to *behavior*, a preliminary weight of evidence is visible. Overall, this picture suggests that mmWaves may affect many biological and health categories that warrant further investigation. Several of these categories have potential implications for public health, e.g., cellular oxidative stress, changes in immune function, genotoxicity, brain/neuronal changes, and cell membrane permeability. In particular, effects have been found in all studies that have investigated oxidative stress [cellular stress due to the over-production of reactive oxygen species and the reduction of oxidative defenses (11)]. Oxidative stress underlies many auto-immune and chronic conditions, such as diabetes, cardiovascular disease, Alzheimer's disease and depression, some of which are becoming an increasing social and economic threat worldwide (12).

The existing epidemiology papers [listed in Supplementary Table 1 (epidem papers)] mainly focus on the effects of occupational exposures, e.g., the occurrence of lymphoma or the reduction of sperm count in radar workers. Of these papers, the majority show effects from mmWave exposures.

### Countries involved in mmWave research

In order to understand where the mmWave research has been performed, the country of origin was extracted from ODEB for all the papers included in this overview. Results showed that a large proportion of the research has been carried out in Russia (23%) and in the US (21%). Some countries have conducted several studies, and these make up a further third of the research: Italy (10%), France (6%), India (5%), Armenia (5%), Japan (4%), and China (3%). Countries that have each conducted only a few studies make up the remaining 23% of the research base.

## Discussion

### An overall trend despite the limited number of studies

Figure 1 shows that the relevant experimental research is minimal, as has been acknowledged in reviews (1, 2, 13). It is thus far too early for scientists to establish any definite theories because the experimental work using mmWaves is limited, there are a large number of end-points and processes to be considered, and for some biological end-points, the evidence appears contradictory. However, Figure 1 also reveals that the overall picture emerging from the existing knowledge base suggests a range of biological effects, some with strong evidence (>90% of studies), that may have potential health implications.

From the existing research, we can draw two conclusions:

1. For scientists, *the understanding of how mmWaves affect biological systems is still in its early stages*, thus there is an urgent need for further focused research to be conducted;
2. For policy makers, *there is enough smoke to suggest the risk of fire*, and therefore there is an urgent need for protective policy.

As Gee has pointed out (14), these two statements are not contradictory. The amount of evidence available in any area of science lies on a continuum from very weak (1–10%) to very strong (90–100%). Scientists require *strong* evidence of causality before laying down a new theory. In the case of the existing evidence for harm from 5G, scientists rightfully maintain that there are *no well-understood causal* links. However, government authorities tasked with protecting the health of humans, animals or plants need only *moderate* evidence as reasonable grounds for concern to enact the Precautionary Principle [e.g., (15)]. With so few experimental studies, but with an overall trend for biological effects, Figure 1 suggests that the current situation is one of *plausible risk*.

While the field of mmWave research has a limited knowledge base, there are early signs of evidence for bioeffects (as described above) that have implications for health. It is interesting to compare the interpretations of this state of affairs made by scientists compared to global policy makers. The science regarding skin is still *insufficient* to *devise science-based exposure limits*, says the scientist Leszczynski and so *precautionary measures should be considered for the deployment of the 5G* (13). In contrast, the industry-linked ICNIRP and the European Union (16) have determined that insufficient evidence provides reassurance of safety. No *evidence of harm* has been misconstrued as *evidence of no harm* [(17), p. 690], allowing the 5G rollout to proceed unfettered.

### Standards compromised

When setting exposure limits, ICNIRP has not addressed the early evidence of biological effects with the potential to cause harm (18), as would be required by a risk management approach. ICNIRP radiation protection philosophy is thus deficient and not in alignment with that of the International Commission on Radiation Protection (ICRP) (19). The ICRP has a clear philosophy of radiation protection based on Justification, Optimization and Limitation.

Under the ICRP global radiation protection code of ethics, where mass exposures of populations are occurring without permission, even mild evidence of harm would be enough to advise governments to give pause to the technology, to consider the potential risks, to commit funds to further research and to enact strict precautions.

These precautions are not being implemented because the early message of plausible risk is unfortunately not being heard, partly due to poor reasoning and partly due to poor communication, as described below.

### Logical fallacies in the communication of science

Along with assessing data quality, researchers can use the tools of reason to assess the quality of statements made in papers. Logical fallacies occur when various methods of argument are used to distort the reasoning, either intentionally or not (20). The art of integrating logical fallacies into communications has been used in the past by selected scientists working for industry, in order to convince the public and policy makers that their products do no harm, e.g., the smoking lobby used such techniques for decades (21). We have found that faulty reasoning has also been used to discuss mmWaves both in the public domain and in the research literature (4). To bring these issues to light and to invite discussion, some of the more frequently used logical fallacies are named in the sections below. These fallacies may not be intentional; e.g., they may be a result of simplifying the message so that the public can digest it. However, it is the responsibility of protection agencies, industry and researchers to ensure that their communications are clear and that fallacies are not inadvertently created when information is delivered to policy makers and to the public.

### Fallacies used in describing millimeter waves

When government agencies or researchers introduce 5G technology as being based on mmWaves which are already in use in airport security screening [e.g., (2, 22)], this can create a “Faulty Analogy”. This type of fallacy occurs when **two** things are alike in one or more ways, but then the incorrect assumption is made that they are necessarily alike in other ways (23). In this case, airport scanners and 5G technologies are similar in one way, in that they both use mmWaves; however, this similarity can lead people to believe that 5G technologies are also just as harmless as they believe airport scanners to be. In reality, the two types of technology are dissimilar in several important ways that are not mentioned in communications: (i) airport body scanners expose people for a few seconds and very infrequently, whereas exposures to 5G technologies occur many times a day throughout a person's lifetime, and (ii) the waveforms used by airport scanners are much simpler and not easily comparable with complex 5G waveforms. Using a Faulty Analogy to introduce mmWaves to the public could prevent consumers from considering any risks or from taking active precautions.

Millimeter waves are also introduced as if they are harmless for the human body. For mmWaves, the critically exposed organs are the skin and sclera of the eyes, and when 5G exposures are being discussed, it is often stated that mmWaves do not penetrate more than a few millimeters into the skin. This creates a “Red Herring” fallacy (23), because it diverts attention toward the less important issue of skin surface tissue, and away from the more important issues of the mechanisms and biological functions of the skin. The facts that are ignored are: (i) In skin research, *penetrates* is a technical term, meaning that two-thirds of the original signal's energy is absorbed. There is still one-third that travels further, into deeper skin layers, nerves and blood. (ii) Skin is rich in nerves that are connected to the central and autonomic nervous systems. (iii) Skin is the body's first line of defense, rich in protective bacteria and part of the immune, waste management, and endocrine systems (13, 24).

There is very limited research into the bioeffects of mmWaves on the skin (13). The endocrine neurotransmitter and cardio systems to which the skin is connected and the critical sclera of the eyes have had a cursory investigation, as shown in Figure 1. However, it is predicted from theoretical models that the skin's sweat gland ducts (SGD) act as helical antennas, which can potentially carry mmWaves much deeper into the body (25, 26). Such deeper penetration has been confirmed, albeit at higher frequencies (94 GHz) (27). There are also predictions that transients from short pulses due to high data rates may create secondary waves called *Brillouin Precursors* that penetrate even deeper into the body, leading to the unwinding of large molecules, cell membrane damage and blood-brain leakage (28). Furthermore, Brillouin precursors do not decay as expected, which can lead to hot spots deep within the body (29). There are further concerns that the rapid pulse trains contained within 5G signals will cause intense hot spots on the skin, resulting in permanent tissue damage (30), and that the current ICNIRP guidelines do not protect against these hot-spots (31).

Altogether, these facts paint a very different picture of plausible risk than does the “Red Herring” statement given in public 5G communications that mmWaves only penetrate a few millimeters into the skin. Fifth and sixth-generation technologies should not be advancing without investigating the above issues, which are currently being ignored.

### Fallacies used in reviews

When mmWave reviews are conducted, several principles are repeatedly used for critiquing experiential design and for dismissing or excluding various papers. However, we have found that several fallacies are present in these arguments, as described below.

#### Exposure principles confuse necessary and sufficient conditions

Quality studies need to report the dosimetry of the exposure signals clearly (i.e., what frequencies were used and what power densities or SARs were measured). Good dosimetry is a *necessary* condition of good reporting. However, it is not *sufficient* to guarantee that the exposures used in the experiment are adequate for testing the hypothesis, for the following reasons.

Real-world 5G signals are complex and variable. First, there are the variable low-frequency pulses (control, pilot, synchronization signals) and modulations being carried on the high-frequency 5G carrier waves. In addition, to send multiple signals simultaneously, many 4G/5G technologies use Orthogonal Frequency-Division Multiplexing (OFDM), which requires extremely high peak amplitudes. These methods of signal transfer create complexities in the waveforms that cannot be fully replicated using simulated signals created by frequency generators. Complex real-world signals are more bioactive (32) and are thus more likely to show bioeffects. Not surprisingly then, experiments that use signal generators are less likely to produce effects, while those that use real-world devices (e.g., mobile phones with, 50, 200, 500, or 217 Hz pulses embedded within the signals) are more likely to produce effects (32). That is, experiments that use real-world signals have a higher power (probability of finding an effect if there is one) than experiments that use simulated signals.

The type of exposure (to real-world devices/signals or to signal generators) thus needs to be a principle for judging the quality of a paper. However, this important principle is often ignored. Instead, a “Confusion of Necessary with Sufficient Condition” fallacy occurs, where a study is acknowledged for reporting the *necessary* dosimetry, but the review does not ensure the inclusion of the more important *sufficient* conditions of the exposure, required to test the hypothesis. This means that studies with lower power are included in reviews and treated as if they are of high quality just because they reported the dosimetry. At the same time, studies with a higher power, that used real-world signals can be dismissed in the review because they do not clearly report the dosimetry [e.g., (33)].

As noted in (32) some reports have claimed that experiments that use a simulated signal from generators are superior because this allows the signal to be controlled in the laboratory experiment. However, this can be a “Red Herring” issue. While highly controlled experiments are to be aimed for, they are not the highest priority if they prevent the experimenter from being able to test the stimulus that is creating the response (which thereby reduces the power of the test).

#### Weakest points rather than strengths highlighted

Reviews also use other “quality of the study” issues to exclude papers or to downplay their results [e.g., (2)]. However, some of these issues are actually examples of the “Straw Person” fallacy, which occurs when the weakest points of an argument are attacked while stronger points are ignored. This fallacy can create a misrepresentation of an opponent's position in order to make one's own argument appear superior. Examples of the “Straw Person” fallacy occur in reviews that use less important issues as grounds to dismiss otherwise relevant and scientifically sound papers. Some examples of “Straw Person” dismissals are given below.

#### No replication or inconsistent results used to downplay results

Due to the low number of mmWave studies, the complexity of available parameter combinations, and given that all the studies are forging new ground, a lack of replication and inconsistencies between studies is to be expected. Moreover, it is well-known that funding bodies and universities do not fund replication studies. Therefore, lack of replication is a “Straw Person” in this emerging field, and to downplay the results of a sound experiment on that basis is fallacious; e.g., Two *studies by a Russian research group have also reported indicators of DNA damage in bacteria; however, these results have not been verified by other investigators* [(2), p. 599].

Collective “Straw Person” dismissal also occurs. For example, Figure 1 shows a range of bioeffects, leading to the suggestion of considerable “smoke” that warrants further investigation of a possible “fire”. In contrast, the range of bioeffects is watered down in (2) by framing them as not yet replicated; e.g., *Although many bioeffects have been reported in many of the experimental studies, the results were generally not independently reproduce*d [(2), p. 600].

#### “Poor methodology” has several meanings

Most experiments can be critiqued for containing some flaw or another; however, flaws occur on a continuum from minor to serious. To accuse a study of a *serious* methodological flaw requires a precise description of that flaw, e.g., the identification of a confounding variable. In contrast, if an experiment includes a noise factor, this is not a serious methodological flaw. The noise factor may weaken the result, by adding more randomness to the measurements, and therefore making it less likely that an effect will be found (i.e., by reducing the power of the test); however, the noise does not fully compromise the study.

Thus, when the term “methodological flaw” is used throughout a review, a logical fallacy of “Equivocation” may occur, because the meaning of this key term has one meaning in one portion of the discussion and then another meaning in another portion of the discussion (23). A concluding summary statement, e.g., that “many of the mmWave papers have methodological flaws”, may then give the impression that all these studies have *major* flaws. In reality, many of the papers could contain non-major issues, such as noise factors and incorrect error bars. Without full explanations, it is impossible to tell if the flaws that papers are being accused of are fatal or non-fatal. We suggest that future reviews avoid a possible equivocation fallacy, by classifying methodological flaws into levels of seriousness, such as high, medium, and low and by giving clear justifications for why each paper is classified as such.

#### Non-linear dose-response misunderstood

Sometimes papers are rejected because they do not show a linear relationship between dose (exposure intensity × exposure duration) and effect. This is an incorrect rejection built on the “Red Herring” assumption that there is a linear relationship between dose and effect for radiofrequencies. This assumption has been countered by research that shows that (i) there are windows of power and frequency that cause harm (34), and (ii) that the human perceptual system has a non-linear response to electromagnetic frequencies (35–37). While linear dose-response models may be appropriate for telecommunications signaling, they are not appropriate for modeling biological responses where feedback mechanisms and adaptive responses occur.

The above examples of inappropriate dismissal of papers in reviews suggest that the credible evidence base for mmWave effects is likely to be larger than stated. To quote Barnes and Greenbaum (38), also cited by Lai (39).

*The evidence that weak radiofrequency (RF) and low-frequency fields can modify human health is still less strong, but the experiments supporting both conclusions are too numerous to be uniformly written off as a group due to poor technique, poor dosimetry, or lack of blinding in some cases, or other good laboratory practices* [(38), p. 2].

#### Conclusions from reviews can be misinterpreted

After dismissing much of the evidence showing effects, as well as reporting the contradictory results, reviews have concluded that there is *no conclusive evidence* of harm. However, an “Appeal to Ignorance” fallacy can occur when the reviewers, the industry, and ICNIRP then give the impression that the statement *there is no harm* must be true because no counter evidence to that conclusion has been found; i.e., *because we have not found conclusive evidence of harm*. This fallacy has the effect of wrongfully shifting the burden of proof away from the one making the claim of no harm (23). In reality, the onus of proof is on industry and government to continue funding research that can enable a better understanding of the effects of mmWaves on humans and the environment.

The above logical fallacies embedded within the analysis and communication of the mmWave science may have resulted in significant omissions of critical studies or incorrect judgements about papers within reviews, making their conclusions unreliable; [e.g., see (4)].

Reviews that contain these fallacies are not a suitable basis on which to build public policy or safety standards.

### Fallacies used in setting standards

Several fallacies are also embedded within the ICNIRP guidelines, for mmWaves as well as other radiofrequencies.

#### Only heating matters

The main fallacy that has been pointed out by many researchers is the “Thermal Only” fallacy, whereby ICNIRP and industry have adopted the position that *only heating can produce important biological or health effects*. This “Red Herring” takes the focus away from research that investigates non-thermal biological and health effects. For example, in the main mmWave literature review of skin effects presented within the current ICNIRP guidelines, a decision has been made to focus on heating effects only [(9), p. 6–8].

#### Averaging is an adequate measure of harm

When ICNIRP assumes that averaging over time and space are effective measures for measuring the level of harm, this is the fallacy of “Slanting” because not all of the evidence available is being used to inform the case (20).

The ICNIRP premise that averaging over time and space is sufficient to calculate harm from exposure is deficient in realism in several ways. First, the statistical use of an average assumes an underlying normal distribution, which is not the case for complex telecommunications signaling. Moreover, averages hide potential biophysical effects resulting in a conclusion of no harm overall, even though extreme harm may have occurred for a small portion of tissue [see (18, 30)].

### Authority uncertain

The fallacy of “Appeal to Authority” occurs when claims are believed because they are made by alleged authorities, but not all of the following are true: (i) they are making claims within their field of expertise, (ii) they are presenting facts about which there is some agreement, and (iii) they can be trusted (23). While bodies like ICNIRP and the WHO International EMF Project are given formal authority, other researchers have criticized them for being a small-self referencing group (40) with no dissenting voices (41). These bodies present one consistent message: that there is no evidence of harm from radiofrequencies, including mmWaves. In contrast, hundreds of scientists around the world with concerns for safety have appealed to the European Union for a moratorium on the 5G rollout (42, 43). Because there is no clear agreement on the facts, to assume an ultimate voice of authority on this topic is fallacious.

Furthermore, some expert scientists researching in this field have links with industry; therefore, conclusions from their papers need to be treated with caution. This is because industry can influence the science (44). For example, industry-funded research for UHF studies (including when partnered with government or military, public trusts, private foundations and institutions) was found to typically use short-term, single one-off exposures created by signal generators, to predominantly expose cell lines (*in vitro*) rather than live animals (*in vivo*) and to avoid epidemiological studies (45). These design decisions have resulted in studies that do not provide insights into potential health effects associated with multiple long-term, real-world exposure scenarios.

**Figure 2:**
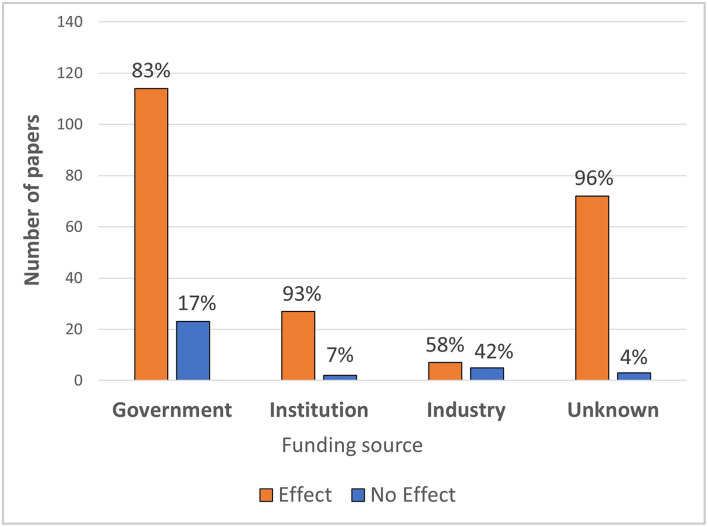
The relative proportion of “Effects” and “No Effects” outcomes from studies according to the funding source.

Similar to Huss et al. (46), an analysis of mmWave studies demonstrates how industry funding influences outcomes. Industry funded mmWave studies have produced a lower overall proportion of “Effect” outcomes, compared to government-funded and institution-based studies (see Figure 2).

## Conclusions

The potential long-term health risks from global EMF continue to rise as exposures in the built environment increase in time and density. Mankind has chosen to base the justification for this rollout on shaky foundations, where there is minimal understanding of the impact of new radiofrequencies being introduced into the environment on long-term human and planetary health.

The evidence presented above suggests that there are credible risks of biological interference effects for frequencies planned for 5G, occurring well-below ICNIRP reference limits. Given the ubiquitous and often non-consensual nature of man-made wireless radiation exposures, the presence of even a small number of significant bioeffects requires follow up with more focused research.

The communication of existing investigations has not been fully clear or transparent. It is the responsibility of government review panels, regulatory bodies, scientists, public advocates, industry and policy makers to clearly communicate the research and its implications, so as to ensure that no fallacious conclusions can be drawn. If these are allowed to continue, both those delivering the message and the unsuspecting billions using their new 5G devices may be led in a direction that places global public and environmental health at risk.

The mmWave evidence base that has been made visible in this article suggests that plausible health effects cannot be ruled out, and that urgent action is needed on two fronts:

1. Further sound scientific research, *done carefully, using the best laboratory practices and sufficiently large samples to produce significant results*, funded and overseen by trusted bodies with appropriate expertise (38).
2. Precautionary actions to be taken by policy makers *via* use of *risk aversion* strategies such as the actions recommended in an EU commissioned report [(47), p. 152–153]. Risk aversion constitutes good leadership.

The limitations of scientific knowledge imply moral courage in taking precautionary action in time to avert harm [(17), p. 687].

## Author contributions

All authors listed have made a substantial, direct, and intellectual contribution to the work and approved it for publication.

## Footnotes

^1^ODEB is a free research tool Available at: https://a037613.fmphost.com/fmi/webd/Research_Review_V4.
